# Bovine tuberculosis in cattle slaughtered at Addis Ababa abattoir in Ethiopia and workforce awareness of zoonotic risk

**DOI:** 10.1371/journal.pone.0321844

**Published:** 2025-05-28

**Authors:** Fahmi Mohammed Ahmed, Musse Girma, Getnet Worku, Tekalign Tadesse, Girmay Medhin, Simon J. Waddell, Balako Gumi

**Affiliations:** 1 Aklilu Lemma Institute of Pathobiology, Addis Ababa University, Addis Ababa, Ethiopia; 2 College of Veterinary Science, Mattu University, Mattu, Ethiopia; 3 Global Health and Infection, Brighton and Sussex Medical School, University of Sussex, Brighton, United Kingdom; National Research Centre, EGYPT

## Abstract

**Background:**

Bovine tuberculosis (bTB) is endemic and of zoonotic importance in Ethiopia. Despite this, there is limited recent information on the prevalence of bTB in cattle slaughtered at abattoirs. This study aimed to estimate the prevalence of bTB in cattle slaughtered at Addis Ababa municipality abattoir based on tuberculous lesions and region of difference (RD4) deletion typing and to assess the current practice and the awareness of occupational workers to zoonoses.

**Methods:**

A total of 502 cattle slaughtered at the municipality abattoir (260 in the day shift and 242 in the night shift) were included in this cross-sectional study. Data collection and laboratory investigations included postmortem examination, culture and bacteriological examination, molecular characterization of positive isolates using region of difference (RD4) deletion typing and spoligotyping. Knowledge of zoonotic infection risk and practices was investigated through a questionnaire administered to 58 abattoir workers and 58 butchers.

**Results:**

Based on postmortem examination, bTB suspected lesion was identified in 4.58% of cattle and it was significantly associated with, the age, breed and body condition of the animals. The detection of tuberculosis lesions during the night shift of the slaughter program was 1.54 times that of the day shift which is not statistically significant but warrants future study with a larger sample size. The gross lesions were predominately found in the lung and associated lymph nodes (65.5%). Of the 23 bTB suspected tuberculous lesions cultured, 11 (47.83%) tissue samples were culture-positive, and four isolates were RD4-positive, identifying *M. bovis*. Spoligotyping patterns were also effectively detected in four isolates. The observed spoligotype patterns were two SB1477 strains, and SB1176 and SB0133 strains. In the questionnaire survey, 79.31% of abattoir workers were aware of bTB, however, 93.10% of butchers did not know of bTB and understood less about preventing cross-infection.

**Conclusion:**

Bovine tuberculosis is evident in cattle reaching the abattoir in Addis Ababa with nearly similar gross lesion pathology-based prevalence of bTB to prior findings reported from the same abattoir a decade ago. This suggests that despite efforts to control the disease in cattle, the prevalence remains largely unchanged. Although statistically non-significant, the trend showing higher odds of detecting tuberculous lesions during the night suggests a need for improved meat inspections during the night shift and the need for larger future studies.

## Introduction

Bovine tuberculosis (bTB) is a chronic disease that primarily affects cattle and yet also infects a diverse range of hosts, including other domesticated animals, wildlife species, and humans [1]. *Mycobacterium bovis* is the principal cause of bTB, and it is transmitted from cattle to humans primarily through the consumption of contaminated or raw milk and meat [2]. Bovine tuberculosis negatively affects both domestic and international economies, has an ecological impact owing to wildlife transmission, and is a public health concern due to its zoonotic importance. Likewise, it has a significant impact on milk and meat output, as well as production; hence it is of economic importance [3].

Bovine tuberculosis is a major public health issue in developing countries that lack the financial capacity to implement costly test and slaughter procedures. It is a chronic, primarily respiratory disease that is difficult to diagnose, despite the fact that emaciation, lack of appetite, chronic cough, and other indicators of pneumonia may appear in cattle at the late stages of the disease [3]. Bovine tuberculosis is characterized by the development of granulomatous lesions during meat inspection activities on cattle carcasses in slaughterhouses, these lesions are identified mostly in the lung and associated lymph nodes, as well as gastrointestinal tract (GIT) organs and related lymph nodes [4]. Mycobacteria can also cause systemic infection, spreading throughout the host to other organs. The most common way for cattle to become infected with *M. bovis* is through aerosol exposure, likely causing lung disease, however, infection can also occur through ingestion of contaminated materials [5].

Human tuberculosis (TB) of animal origin, especially from *Mycobacterium bovis*, is a serious public health problem. Before obligatory pasteurization, *M. bovis* was responsible for one-fourth of all pediatric TB cases in developed nations [6]. However, the incidence is now estimated to be ~1 in 100,000 people each year [7,8]. In developing countries, 10% to 15% of human TB until the end of the 1990s was believed to be caused by *M. bovis* [6,8]. In a more recent study, only 2.8% of human TB cases in Africa were caused by *M. bovis* [7]. Human TB of animal origin has been a longstanding problem in Ethiopia [9–12]. Most recently, *M. bovis* was isolated from 2.4% TB patients in central Ethiopia [13]. This may be linked to the high risk of zoonotic TB incidents as the result of shared shelters between cattle and humans, and the habit of consuming raw milk and meat [8,14].

Several abattoir-based prevalence studies in Ethiopia in the last decade [11,15–17] have reported bTB in cattle based on the presence of TB-like lesions, with an estimated average prevalence of 5.57%. Earlier Ethiopian studies isolated *M. bovis* from TB lesions in cattle, spoligotyped, and identified strain types [10,15,18–21]. However, genotyping of *M. bovis*, which could indicate strains circulating in cattle slaughtered at abattoir, has been inadequate. Up to date epidemiology of bTB in cattle destined for market and a current understanding of bTB awareness in workers in the meat industry is required for effective control strategies and to improve public health. This study estimated the prevalence of bovine tuberculosis in cattle slaughtered at the municipal abattoir in Addis Ababa, using molecular methods to define the causative agent, and assessed abattoir workers’ awareness to gauge industry knowledge of this disease and risk of zoonotic infection.

## Materials and methods

### Context of the country and description of study abattoir

Ethiopia is a country of people with various religions and diverse cultures. Orthodox Christianity is among the major religions that have a significant number of followers and they practice fasting (*i.e*., abstinence from animal products) twice a week (*i.e*., Wednesday and Friday), about two months fasting before Christmases, about 50 days fasting before Easter and several other fasting times ranging from three days to 15 days. Followers of other religions also have their specific cultural practices about animal product use. Holidays in Ethiopia are generally known for the significant consumption of meat. These practices have implications on which days to investigate animal product-related issues. The current study was conducted at the Addis Ababa Municipality Abattoir located in Addis Ababa city. The city is located at a latitude and longitude of 9°14’8“N and 38°44’24”E, respectively, with an average altitude of 2500 meters above sea level. It is structured into 11 sub-cities, namely, Addis Ketema, Akaky Kaliti, Arada, Bole, Gullele, Kirkos, Kolfe Keranio, Lideta, Nifas Silk-Lafto, Yeka and Lammi Kura. Cattle originating from different parts of the country are brought for slaughter at this abattoir. The abattoir supplies cattle, sheep, goat and swine meat and associated edible organs to Addis Ababa residents on a daily basis. However, the supply varies across days of a week, throughout different months, and during selected annual holidays. The municipality abattoir accounts for 85% of total meat production in Addis Ababa. Kara-Alo Abattoir and Abattoirs in the Oromia regional state near Addis Ababa cover the remaining 15% of the meat demand of Addis Ababa residents [22].

### Study design and study population

A cross-sectional study was conducted from May 2021 to July 2022 to determine the prevalence of bTB based on gross lesion identification and to define the molecular characteristics of bTB isolates in cattle slaughtered at the Addis Ababa municipality abattoir. The study also assessed the practice and awareness of abattoir workers and butchers with respect to potential bTB zoonoses. Cattle brought to the abattoir during the study period for slaughter were eligible for the study irrespective of their age, sex, breed, body condition scores and origin. All consented abattoir workers and an equivalent number of butchers completed the questionnaire survey.

### Sample size determination and sampling method

The total sample size of animals included in this study was determined using Epi Info™ 7 by choosing the population survey option [23] with the expected 5% prevalence of bTB in Addis Ababa from a previous study [16] with 95% confidence interval and 2% margin of error. Accordingly, the required minimum sample size was 456 cattle. However, considering the precision and representativeness of data, a 10% non-response that covers refusals and various sources missingness factor (n=46) was considered. Hence, a total of 502 animals were included in this study.

Animals for the study were recruited using a systematic random sampling technique in which the starting point was chosen at random on every sample collection day. Individual animals on a given day were taken at regular fixed intervals (every k^th^). The sampling interval for a given day was determined by dividing the total number of animals brought for slaughter within that day by the estimated daily sample size, which was 5–10 animals based on the capability of the researcher to assess. A stratified random sampling approach where the animal pool was divided into subgroups based on their age was used; especially during the holidays when the number of animals slaughtered significantly increased. Eventually, the number of animals to be assessed each day was proportional to the number of animals brought for slaughter. For the questionnaire survey, participants were selected depending on their occupational closeness to the animals and consent for participation. Accordingly, 58 abattoir workers who gave consent and an equal number of butchers, a total of 116 individuals were enrolled in the study. A stratified random sampling technique was employed for the butchers to select the appropriate representative data from the five sub-cities (Bole, Kirkos, Yeka, Gullele, and Lideta) nearly fully supplied by the municipality abattoir.

### Abattoir survey

#### Antemortem and postmortem inspection.

The antemortem inspection of each study animal was conducted according to the antemortem inspection protocol developed by a previous study [24]; particularly abnormality in gait, any visible discharges from natural orifices, lesions on the body and body conditions were examined Factors including age, breed, body condition score (BCS) and sex were considered as possible risk factors for prevalence of bTB.

The postmortem examination was carried out following the OIE and Meat Inspection and Quarantine Division of the Ministry of Agriculture method [25–27]. Accordingly, inspection of any visible gross lesions, and palpation, followed by incision of the organs and lymph nodes, was conducted to examine for the presence of tuberculous lesions.

#### Specimen collection and transportation.

For suspected tuberculous lesions, approximately 2 mm thick tissue specimens were collected carefully and placed into sterile universal tubes containing 0.9% sterile saline. The samples were stored in an ice box containing ice packs and transported to the Aklilu Lemma Institute of Pathobiology for culture and further bacteriological analyses. The samples were stored at -20°C before processing.

#### Culture and identification of mycobacteria.

The processing of tissues with suspected lesions and culturing for isolation of mycobacteria was carried out on a Lowenstein-Jensen (LJ) medium in accordance with the guidelines of the Office of International des Epizooties [27]. Samples from a single case were pooled in one medium and cultured. The cultures were incubated in a slanted position aerobically at 37°C for 1 week and then in an upright position for 7–10 weeks. The tubes were tightly closed to avoid desiccation, and slopes were examined for macroscopic growth at weekly intervals for the presence of mycobacterial colonies [27].

Initial identification of mycobacterial species was based on the rate of growth, pigment production, and colony morphology as described in [27]. When visible colonies were observed, Ziehl Nielsen staining was performed for the quality control purposes to confirm the presence of viable acid-fast bacilli (AFB).

#### Preparation of bacteria for molecular characterization.

AFB-positive colonies were harvested for molecular characterization by scraping growth from the LJ medium slopes and suspending them in 300 μl sterile distilled water. The bacteria and the distilled water were mixed thoroughly and the mixture was heated at 80°C for 45 minutes in a shaking heat block. The heat-killed isolates were stored at -20°C.

#### Molecular typing of the isolates.

**Region of difference (RD4) deletion typing:** Genomic DNA was extracted from the heat-killed isolates and RD4 deletion typing was performed as described by [28]. DNA from culture-positive samples was used as a PCR template. Genomic DNA from *M. bovis* BCG and H37Rv was used as positive controls for *M. bovis* and *M. tuberculosis* respectively, and distilled water was used as a negative control. In brief, the amplified PCR product was analyzed using 1.5% agarose gel in 1x TBE buffer; ethidium bromide at a ratio of 1:10, with a 100 bp DNA ladder and orange 6x loading dye for gel electrophoresis. The gel was visualized using a UV-Trans-illuminator. The presence of RD4 (that is intact in *M. tuberculosis* and *M. africanum*) generates a PCR product size of 335 bp (RD4 intF + RD4flankR); the absence of RD4 (identifying *M. bovis* isolates away from other *M.tb* complex mycobacteria) generates a product size of 446 bp (RD4flankF + RD4flankR).

**Spoligotyping:** Spoligotyping was conducted following the protocol of the previous study [29]. Briefly, the amplified DNA was hybridized to inter-DR spacer oligonucleotides covalently bound to a membrane. Reference strains of *M. tuberculosis* H37Rv and *M. bovis* 2122/97 were used as positive controls, whereas sterile water was used as a negative control. Hybridization was performed by incubating the membrane for 1h at 60°C. Hybridized DNA was detected by incubating the membrane in enhanced chemiluminescence (ECL) detection fluid for 2 minutes and visualized by exposing it to a light-sensitive ECL film for 20 minutes. The X-ray film was then analyzed by recording the presence or absence of signals at the sites of the individual probes [30].

### Questionnaire survey

A semi-structured pre-tested questionnaire was completed by 58 consented abattoir workers and the equivalent number of butchers to assess awareness towards bTB and the possible risk of zoonotic transmission of bTB. The survey included questions on practices in the abattoirs, which could have an impact on the transmission of bTB to humans, knowledge regarding bTB, and specific questions regarding the transmission modes of disease.

### Data management and statistical analysis

Data analyses were conducted using STATA^®^ Version 15.1 (Stata Corp, USA). The results from the study were summarized using descriptive statistics. A percentage was used to express the prevalence of animals with tuberculous lesions and the frequency of anatomical location. Multivariate logistic regression analysis was conducted to quantify the degree of association between explanatory variables (age, sex and breed of the animals) and the prevalence of bTB. The strength of association was expressed as odds ratio (OR) with corresponding 95% confidence intervals. A p-value of <0.05 was considered as indicative of a statistically significant association. For mycobacterial strain differentiation, spoligotype patterns were converted into binary code and searched in the online spoligotype database https://www.mbovis.org/ to determine the international Spoligotype Bovis (SB) number; the results were compared with already existing designations in the international spoligotyping database [31]. The questionnaire was collected and summarized using the Kobo Toolbox [32] and the results were expressed in terms of frequency and percentages.

### Ethical approval

Ethical clearance was obtained from the Institutional Review Board (IRB) of Aklilu Lemma Institute of Pathobiology, Addis Ababa University. The research work was conducted according to the Office of International Epizooties (OIE, 2018) principles for the use of animals in research and education. The study is reported according to the ARRIVE guidelines [33] to the extent applicable. For the questionnaire survey involving human participants, verbal informed consent was obtained from each participant, after the study details were explained and they were provided the opportunity to ask any questions. The consent was documented by recording participants’ response as either ‘yes’ to proceed and ‘no’ to stop, ensuring clear documentation of their decision and this was approved by the IRB. Confidentiality was maintained by using codes in place of participants’ names.

## Results

### Slaughtered cattle characteristics and prevalence of gross tuberculous lesions

A total of 502 slaughtered cattle were examined in this study; all of which were normal in gait, no visible discharges from natural orifices, and no visible lesions on the body. Inadequate attention from meat inspectors during the ante-mortem stage is noted. Of the total slaughtered animals, 431 (85.86%) were males and 414 (82.47%) were local breeds with a higher proportion of adults 326 (64.94%). The BCS showed 246 (49%) medium body conditioned (Table 1). Based on the postmortem examination, clinically suggestive TB lesions were detected in 23 animals resulting in an overall lesion prevalence of 4.58%. The prevalence of tuberculous lesions was significantly associated with age, breed, and body condition of the animals. The odds of detecting TB lesions during the night shift were 1.54 times that of daytime screening, although this difference is not statistically significant (Table 1).

**Table 1:** Association of gross tuberculous lesion prevalence of bovine tuberculosis with different risk factors for cattle slaughtered at Addis Ababa municipality abattoir.

Variables	Category	No. of animals examined (%)	Positive for TB lesion (%)	Adjusted OR (95% CI)	P-value
**Age**	Young	62 (12.35)	1 (0.20)	1.07(0.12–9.25)	
	Adult	326 (64.94)	9 (1.79)	Reference	0.003
	Old	114 (22.71)	13 (2.59)	4.97(1.87–13.19)*	
**Sex**	Male	431 (85.86)	14 (2.79)	Reference	
	Female	71 (14.14)	9 (1.79)	2.25 (0.75–6.73)	0.144
**Breed**	Local	414 (82.47)	12(2.39)	Reference	
	Cross	88 (17.53)	11(2.19)	3.32 (1.17–9.45)*	0.033
**Body conditions core**	Good	177 (35.26)	2 (0.40)	Reference	
	Moderate	246 (49.0)	7 (1.39)	2.45 (0.49–12.25)	<0.001
	Poor	79 (15.74)	14 (2.79)	17.70 (3.74–83.74)*	
**Sample collection time**	Day time	260 (48.21)	9 (1.79)	Reference	
	Night time	242 (48.28)	14 (2.78)	1.54 (0.57–4.187)	0.218

*Indicates statistically significant association (*P*<0.05) and an odds ratio >1.

### Distribution of gross tuberculous lesions

**Fig 1:**
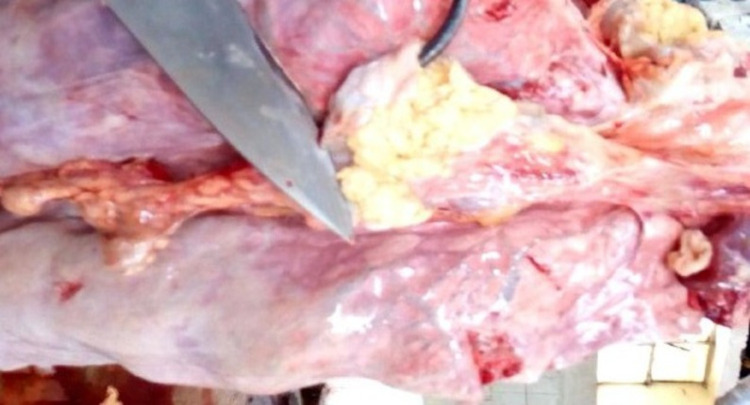
Tuberculous lesions detected in cattle slaughtered in the Addis Ababa municipal abattoir. Granulomatous lesion from a mediastinal lymph node.

**Fig 2:**
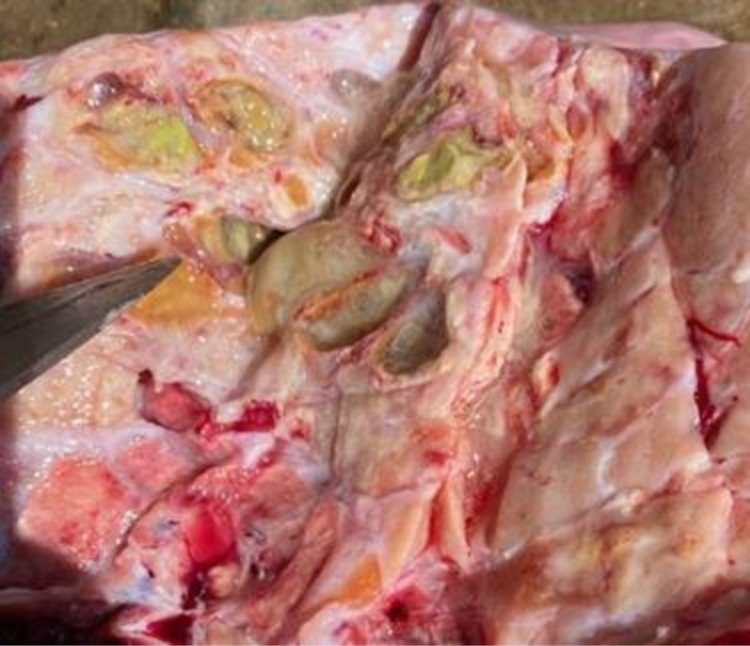
Tuberculous lesions detected in cattle slaughtered in the Addis Ababa municipal abattoir. Tuberculous lesions in lung tissue.

Tuberculous lesions characterized by central round, oval, or irregular, often coalescing areas of caseous necrosis and calcification were observed in the lymph nodes and lungs of the slaughtered cattle (Figs 1 and 2). Gross lesions suggestive of bTB were predominately detected in lung and associated lymph nodes (65.5%), followed by head-associated lymph nodes (17.2%) and then mesenteric lymph nodes (10.3%) (Table 2).

**Table 2:** Distribution of tuberculous lesions in different anatomical sites and their respective frequency of occurrence in cattle slaughtered at Addis Ababa municipality abattoir.

Anatomical Site	Affected Organ/Lymph node	Number (%)
**Head Associated**	Retropharyngeal LN	3 (10.3)
	Mandibular LN	2 (6.9)
**Lung and Associated Lymph nodes**	Lung	9 (31)
	Bronchial LN	6 (20.7)
	Mediastinal LN	4 13.5)
**Liver and GIT Associated LN**	Liver	1 (3.5)
	Mesenteric LN	3 (10.3)
**Superficial LN**	Prefemoral LN	1(3.5)

### Mycobacterial culture and microscopy

Two lesions in six animals were pooled into a single culture medium for culturing. Out of 23 bTB suspected tuberculous lesions cultured (with six samples pooled), there was mycobacterial growth in 11 (47.83%) tissue samples on LJ culture medium ( and 4). Colonies were isolated at a higher frequency from sodium pyruvate-enriched LJ medium, 6/11 (54.5%) than glycerol-supplemented LJ medium, 2/11 (18%). The remaining 27.4% (3/11) were identified from both LJ media formulations. From the eleven cultures positive, six were AFB positive by microscopy.

### Molecular characterization of the isolates

PCR RD4 analysis of the six AFB-positive isolates resulted in four positives for *M. bovis,* with 446 bp RD4 band sizes. The *M. bovis*-positive samples were obtained from lung and bronchial tissue and retropharyngeal and mediastinal lymph nodes. The remaining two AFB-positive isolates did not show any signal suggesting that they might be non-tuberculous mycobacteria (NTM) (Fig 5).

**Fig 3:**
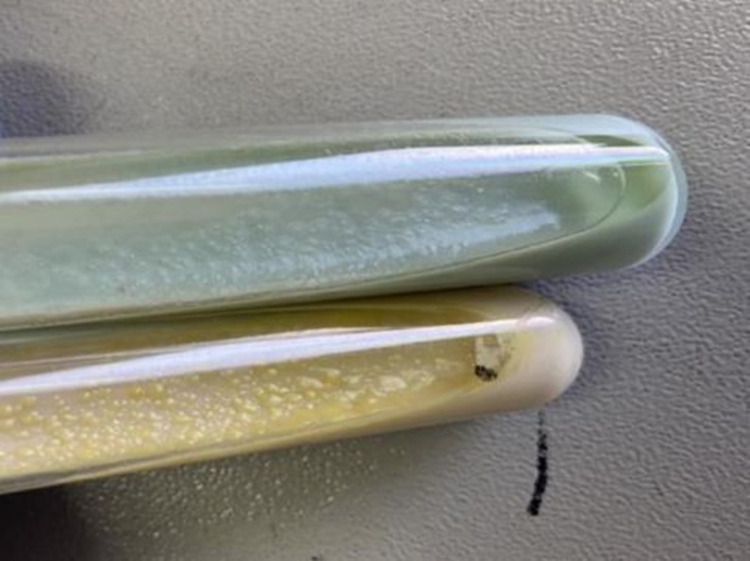
Mycobacteria growth on LJ media. Growth of mycobacteria on LJP and LJG media.

**Fig 4:**
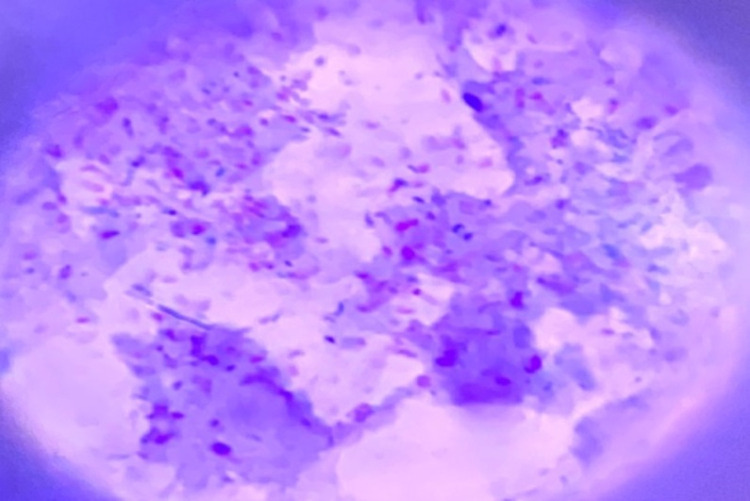
AFB positive mycobacteria by microscopy. 1000× magnification of AFB microscopy showing AFB positive red rod-shaped mycobacteria.

**Fig 5:**
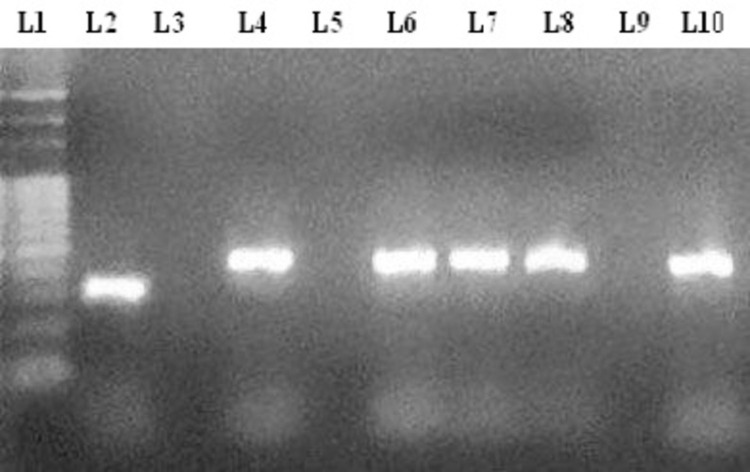
Gel electrophoresis of PCR products from RD4 typing of the AFB positive isolates. L1: DNA ladder; L2: *M. tuberculosis* H37Rv 335 bp control, L3: Distilled water as a negative control, L4: *M. bovis* BCG DNA as positive 446 bp RD4 control, L5-L10: PCR amplicons of AFB positive isolates identifying *M. bovis* in L6, L7, L8, and L10.

The AFB-positive isolates were further characterized using spoligotyping. Spoligotyping patterns were effectively detected in four (36.3%) isolates which were also RD4 PCR positive. Two of the isolates had the same spoligopattern while the other two isolates were genetically different. The molecular analysis of the mycobacterial strains identified three different spoligotypes in the https://www.mbovis.org/ database (Table 3). The observed spoligotype patterns were two SB1477 strains, SB1176 and SB0133. The spoligotypes lacked spacers 3–7, 9, 16 and 39–43 which is a characteristic of *M. bovis*, and confirmed that these cattle had *M. bovis*-derived bTB disease.

**Table 3:** Spoligotype patterns of *M. bovis* isolates from cattle slaughtered at the municipal abattoir.

Isolate ID	Octal number	Binary Code	SB number
***M. tb* H37Rv**	777777477760771	■■■■■■■■■■■■■■■■■■■□□■■■■■■■■■■■□□□□■■■■■■■	SIT451
**Water**	–	□□□□□□□□□□□□□□□□□□□□□□□□□□□□□□□□□□□□□□□□□□□	–
**BCG**	676773777777600	■■□■■■■■□■■■■■■□■■■■■■■■■■■■■■■■■■■■■■□□□□□	SB0120
**A-056**	402773777757600	■□□□□□□■□■■■■■■□■■■■■■■■■■■■■■■□■■■■■■□□□□□	SB1477
**A-121**	402773777757600	■□□□□□□■□■■■■■■□■■■■■■■■■■■■■■■□■■■■■■□□□□□	SB1477
**A-361**	602773761000200	■■□□□□□■□■■■■■■□■■■■■■■□□□■□□□□□□□□□□■□□□□□	SB1176
**A-439**	602773777777600	■■□□□□□■□■■■■■■□■■■■■■■■■■■■■■■■■■■■■■□□□□□	SB0133

### Assessment of awareness and practice towards bTB in meat industry workers

The majority of abattoir worker respondents were aware (79.31%) of bTB. However, nearly all butchers (93.1%) didn’t know about bTB. There were significant differences in the proportion of abattoir workers (81.03%) and butchers (10.34%) who understood that bTB may transmit from cattle to humans and vice versa. Most of the abattoir workers (74.14%) had encountered TB-like lesions before. Unlike butchers, almost all abattoir workers knew about routes of zoonotic infection and knew how to prevent zoonotic TB (Table 4).

**Table 4:** Assessment of practice and respondents' awareness of bTB.

Knowledge of bTB and abattoir practice assessed using questionnaires	Respondents out of 58 (%)	
**Abattoir Workers**	**Butchers**
Had awareness of bovine tuberculosis (bTB)	46 (79.31)	4 (6.90)
Ante-mortem examination is done regularly in the abattoir	31 (53.46)	*
Post-mortem examination is done regularly in the abattoir	58 (100)	*
Know what tuberculous lesions look like	30 (51.72)	4 (6.90)
Encountered TB-like lesions during their work	43 (74.14)	0 (0.00)
How often do you encounter TB-like lesions in the abattoir?		
*Frequently*	11(18.96)	*
*Sometimes*	10 (17.24)	*
*Rarely*	35 (60.34)	*
*Never*	2 (3.45)	*
Aware that cattle can transmit bTB to humans and vice versa	43 (81.03)	6 (10.34)
Knew the consumption of raw animal products possible exposure to bTB	46 (79.31)	6 (10.34)
Consume raw and/or undercooked meat	26 (44.82)	52 (89.66)
Drink raw or soured milk	34 (58.61)	26 (44.83)
Think the habit of raw meat consumption is increasing	*	35 (60.30)
What methods can be used to reduce transmission of bTB from cattle to humans and vice versa?		
*Avoiding close contact*	42 (72.40)	6 (10.34)
*Regular and strict meat inspections*	58 (100)	46 (79.31)
*Avoiding suspected animals to pass for consumption*	58 (100)	29 (50.00)
*Avoid consumption of raw meat and milk*	47 (81.0)	20 (34.48)
*Avoid sharing of shelter with animals*	39 (67.30)	2 (3.45)

*Not applicable

## Discussion

Bovine tuberculosis, caused by *M. bovis*, has been recorded in animals in most developing nations, albeit the scale is difficult to determine because surveillance and control measures are frequently insufficient or nonexistent [16]. Detection of tuberculous lesions in cattle in Ethiopia during abattoir examination is a common procedure. In the present study, detailed postmortem examination resulted in a prevalence of lesions indicative of bTB as 4.58%, which is consistent with previous studies across different regions and abattoirs in Ethiopia with reported prevalence rates ranging from 4.3% to 5.7% [16,34–36] and lower than other abattoir-based studies with a reported range of prevalence from 13.2% to 22% [37,38]. However, lower prevalence has also been reported elsewhere with a range of 1.1% to 2.7% [39,40]. The higher prevalence reported in our study might be due to the time period of the study and breed of animals that are slaughtered in the abattoirs as there is a high number of dairy farms around Addis Ababa and cross-breed cattle are slaughtered in the abattoir. The crossbreeds are relatively more susceptible to bTB infection [26]. It might also be due to the higher local burden of the diseases in a central area of the country [41]. The lower prevalence reported in this study might be due to the smaller sample size in our study and the frequency of collection of non-tuberculous lesions in previous studies. It could also be due to the management and production systems of the slaughtered cattle, as an intensification of the dairy sector in any of the regions may potentially contribute to higher prevalence rates [41].

The burden of bTB in this study was highest among older age group cattle compared to the adult and young age groups, which is in agreement with the reports of municipal abattoirs in Addis Ababa, Dilla, Butajira and Gondar [16,42–44]. This finding is logical; as the age of cattle increases, the probability of acquiring TB infection also increases which can be explained by the declining protective capability in aging animals [45]. The chronic nature of bTB, stress, malnutrition and immunosuppression all increase with age, which contributes to higher susceptibility to disease in older animals [46]. Poor body condition of the animals was one of the factors significantly associated with increased bTB prevalence in the current study. This has been observed in previous reports [43,47] and could be related to the extensive lesions and wasting of the body caused by the chronic nature of bTB disease. The gross tuberculosis lesions were most frequent in the lung and associated lymph nodes, followed by lymph nodes of the head and mesenteric lymph node. Similar distributions of lesions have been reported elsewhere [44,48] alongside a lower prevalence of gross tuberculosis lesions compared to the current study [34,36]. A higher prevalence of lesions has also been described [38,40], highlighting the variation of disease across regions. Detection of the majority of tuberculous lesions in the lung and associated lymph nodes suggests that the animals acquired the infection through the respiratory route [34,40,49].

In the present study, the growth rate of suspected mycobacteria was 47.83%. This report concurs with findings by [10] who reported (47%) but has considerable deviance with the previous study by [26], who reported 56%. The lower growth rate in this study could be due to the fact that *M. bovis* grows poorly on standard LJ medium [48]. In addition, the presence of caseous and/or calcified lesions that have no or very few viable mycobacteria may resulted in a poor isolation rate [36]. The current isolation rate is higher than the previous reports [50,51].

RD4 deletion typing confirmed four isolates to be *M. bovis* (with PCR product sizes of 446 bp). The spoligotype analysis of these mycobacterial isolates identified three different spoligotypes (SB1176, SB1477 and SB0133), which have been previously reported in Ethiopia [16,20,21,34] as a cause of bTB circulating in Ethiopian cattle. All three spoligotypes have a unique spoligotype feature (lacking spacers 3–7) which is indicative of members of the *M. bovis* African 2 (Af2) clonal complex that has only been reported in East Africa so far [52]. Isolation of spoligotype SB1477 conforms to the findings of a previous study [16] from the same abattoir. The SB0133 and SB1176 strains were also reported as the most common spoligotype pattern among Ethiopian cattle isolates [12,34,52]. Even though *M. tuberculosis* was not isolated in the current study, previous studies in Ethiopia have reported both *M. bovis* and *M. tuberculosis* strains in cattle and humans [12,15,18], suggesting that *M. bovis* infection could lead to zoonotic TB. *M. bovis* should be considered a possible hazard for human tuberculosis in countries, like Ethiopia, where eating of raw meat and unpasteurized milk is common in the context of high prevalence of bTB without enhanced management efforts to prevent zoonotic events.

In the present study, special attention was focused to determine if the detection of tuberculous lesions was associated with slaughterhouse work shifts. We found that the detection of tuberculous lesions was 1.54 times more frequent in animals slaughtered during the night shift compared to the day shift. However, this is not statistically significant and needs further investigation with a larger sample size to confirm and understand the reasons behind such an observation.

In the workforce survey, 79.31% of abattoir workers and 6.90% of butchers reported having awareness about bTB, which is similar to a previous study [53], and likely reflects the higher exposure of abattoir workers to diseased cattle pathologies. Most of the abattoir workers knew of methods used to reduce transmission of bTB from cattle to humans and vice versa. This good level of awareness about the transmission of bTB is extremely important for the control and prevention of zoonotic bTB. In the current study, 53.46% of respondents said that ante-mortem examination is performed regularly in the abattoir and 44.82% of the respondents knew that raw or undercooked meat is a potential source of infection. However, 67% of the respondents still consume raw beef meat and 60.3% of butchers perceived that the habit of consuming raw meat is increasing. The consumption of raw animal products and a low level of awareness about the zoonotic implications of bTB amongst butchers (and by extension the general public) are factors likely to promote the spread of tuberculosis as zoonosis in Ethiopia.

Animals slaughtered in the municipality abattoir were brought from different parts of the country passing many hierarchies of markets, and this made it difficult to identify the origin. So, the study’s output is limited and doesn’t cover all aspects of epidemiology including the origin of the study animals. A future study with a higher number of samples is also needed to reduce statistical error and provide a full picture of strains circulating in the study abattoir.

## Conclusion

The current study identified bTB in apparently healthy cattle brought for slaughter to the abattoir. Gross lesion pathology-based prevalence of bTB was moderately low (~5%), however, this is broadly the same as in 2009, from a previous study conducted in the same abattoir. This is may be an indication that control methods in cattle are not impacting disease prevalence. The burden of bTB in this study was higher among older poor body conditioned cattle and a majority of the tuberculous lesions were confined to the lung and its associated lymph nodes suggesting respiratory route as the major means of bTB transmission among the cattle population. This study also highlights the requirement for rigorous antemortem and postmortem inspection procedures, especially during the night shift, and the importance of education to raise the meat industry workforce and community awareness about the zoonotic risk of bTB.

## Supporting information

S1 File Full-length gels/blots of Gel electrophoresis of PCR products from RD4 typing of the AFB positive isolates with different exposures. Gel electrophoresis of PCR products from RD4 typing of the AFB positive isolates. (L1: DNA ladder; L2: *M. tuberculosis* H37Rv 335 bp control, L3: Distilled water as a negative control, L4: *M. bovis* BCG DNA as positive 446 bp RD4 control, L5-L10: PCR amplicons of AFB positive isolates identifying *M. bovis* in L6, L7, L8, and L10. Portions of the original blot have been cropped to focus on the relevant data; L1 to L10).
(DOCX)
