# Supplementary material for: Bovine tuberculosis in cattle slaughtered at Addis Ababa abattoir in Ethiopia and workforce awareness of zoonotic risk
Source: PLoS One. 2025 May 28;20(5):e0321844. doi: 10.1371/journal.pone.0321844 (PMC12119005; doi:10.1371/journal.pone.0321844)
Supplement: S1 File — Gel electrophoresis of PCR products from RD4 typing of the AFB positive isolates. (L1: DNA ladder; L2: M. tuberculosis H37Rv 335 bp control, L3: Distilled water as a negative control, L4: M. bovis BCG DNA as positive 446 bp RD4 control, L5-L10: PCR amplicons of AFB positive isolates identifying M. bovis in L6, L7, L8, and L10. Portions of the original blot have been cropped to focus on the relevant data; L1 to L10). (DOCX) [file pone.0321844.s001.docx]

**Full-length gels/blots of Gel electrophoresis of PCR products from RD4 typing of the AFB positive isolates with different exposures**


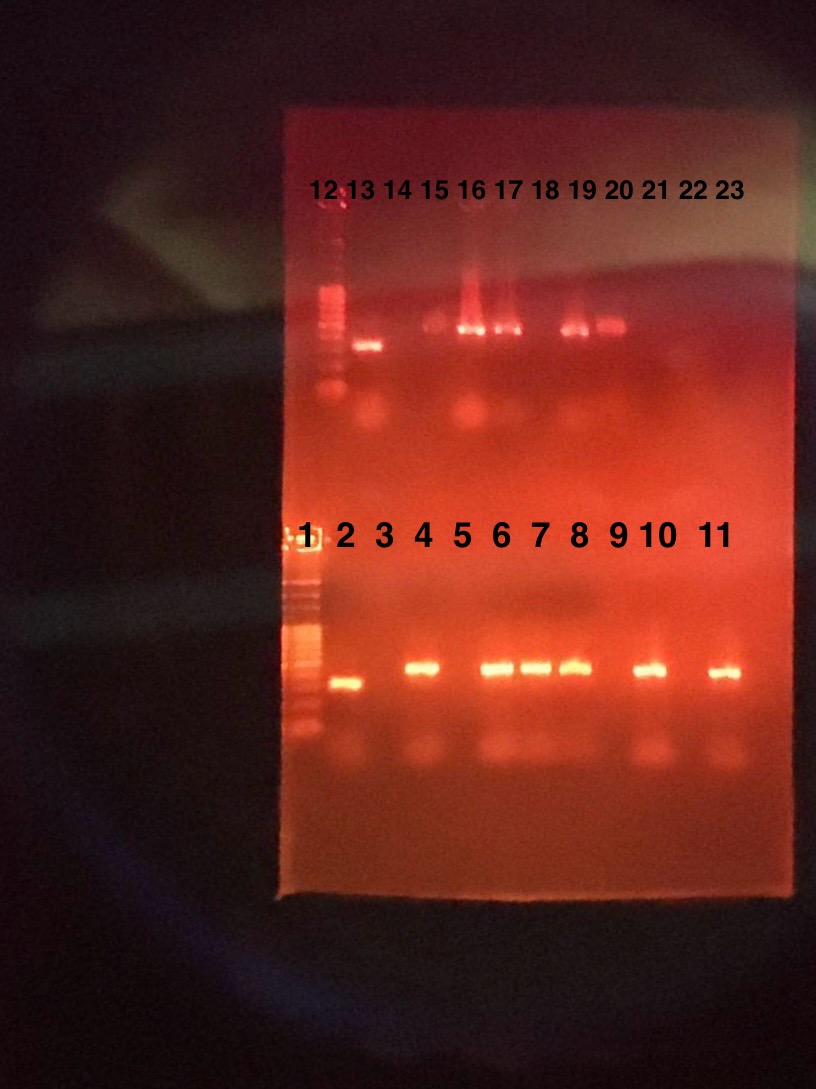

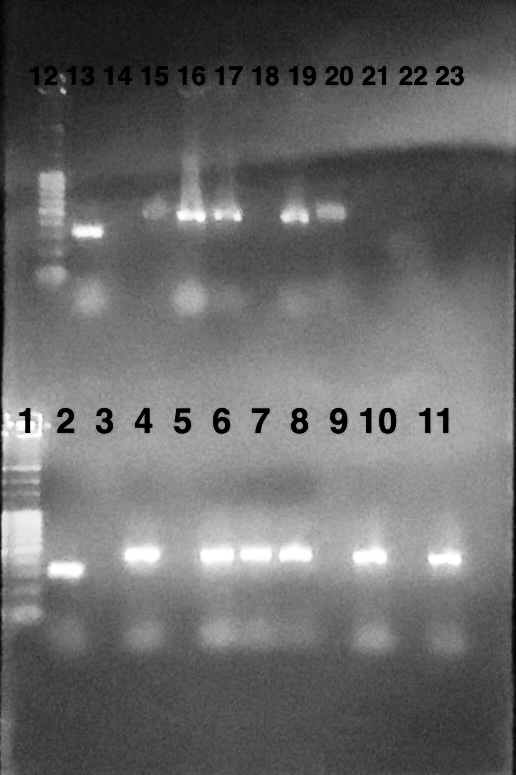


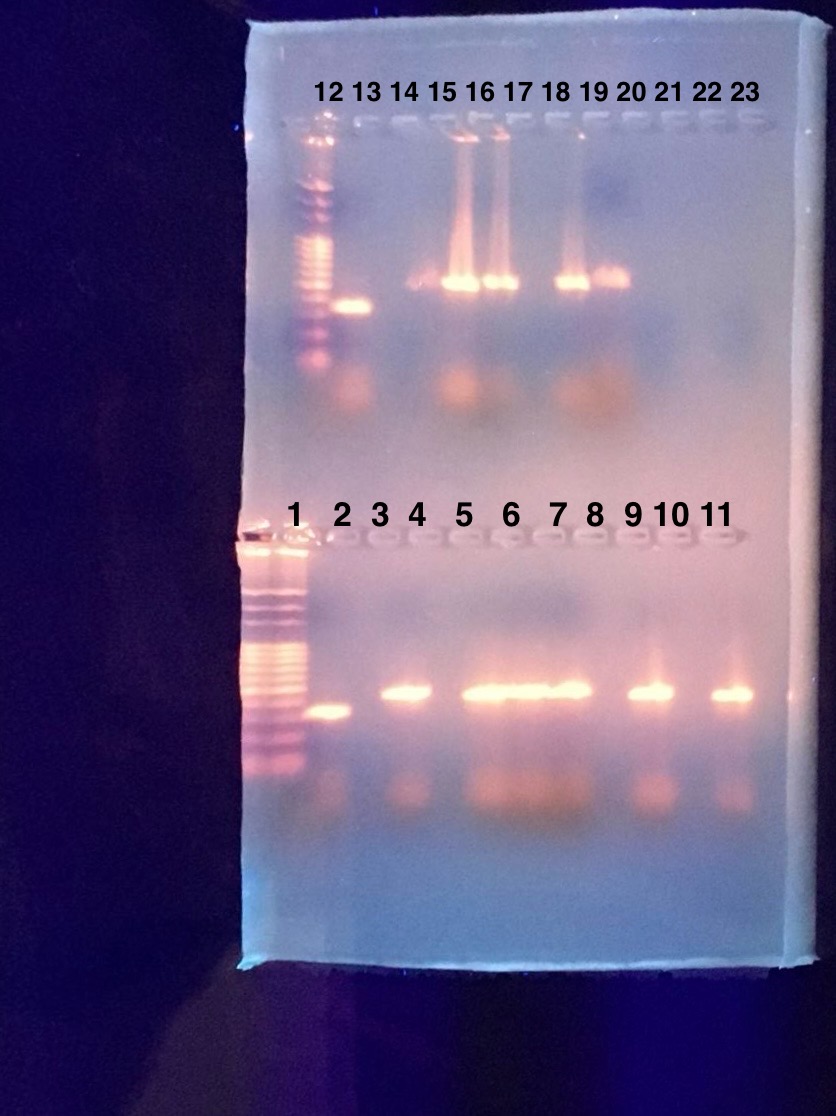

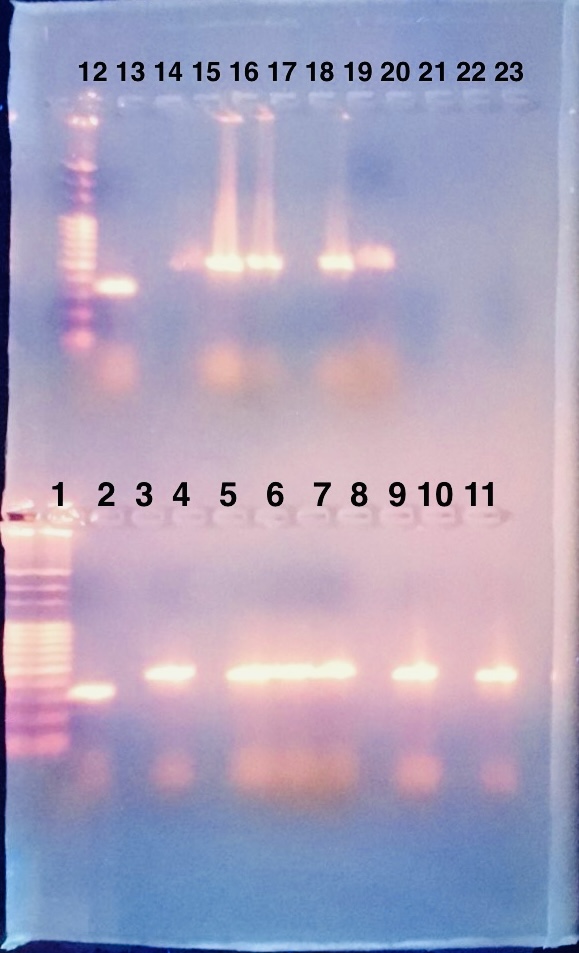


**Supporting Figure: Full-length gels/blots of Gel electrophoresis of PCR products from RD4 typing of the AFB positive isolates with different exposures**

All three figures depict the same full-length gels/blots with varying contrasts representing RD4 (Region of Difference) deletion typing of the AFB (Acid Fast Bacilli) positive isolates from two concurrent research projects displayed on a single gel electrophoresis. To optimize resource usage, we conducted RD4 typing for both projects simultaneously on a single gel electrophoresis. **Our manuscript includes only the relevant portion of the gel (portions 1 to 10).** For clarity, **we have utilized the cropped portion of the second figure (the one on the right from the two above) representing portions 1 to 10.** Additionally, in adherence to the guidelines, we have ensured that the cropped portion is described in the figure legend of our manuscript, as described below.

“Gel electrophoresis of PCR products from RD4 typing of the AFB positive isolates. *(L1: DNA ladder; L2: M. tuberculosis H37Rv 335 bp control, L3: Distilled water as a negative control, L4: M. bovis BCG DNA as positive 446 bp RD4 control, L5-L10: PCR amplicons of AFB positive isolates identifying M. bovis in L6, L7, L8, and L10. Portions of the original blot have been cropped to focus on the relevant data; L1 to L10)”*
